# Assessment of Sensitivity and Profitability of an Intravaginal Sensor for Remote Calving Prediction in Dairy Cattle

**DOI:** 10.3390/s21248348

**Published:** 2021-12-14

**Authors:** Martina Crociati, Lakamy Sylla, Giuseppe Stradaioli, Maurizio Monaci, Alfonso Zecconi

**Affiliations:** 1 Department of Veterinary Medicine, University of Perugia, 06126 Perugia, Italy; lakamy.sylla@unipg.it (L.S.); maurizio.monaci@unipg.it (M.M.); 2 Centre for Perinatal and Reproductive Medicine, University of Perugia, 06126 Perugia, Italy; 3 Department of Agricultural, Food, Environmental and Animal Sciences (DI4A), University of Udine, 33100 Udine, Italy; giuseppe.stradaioli@uniud.it; 4 Surgical and Dental Sciences-One Health Unit, Department of Biomedical, University of Milano, 20133 Milano, Italy; alfonso.zecconi@unimi.it

**Keywords:** GSM, remote calving alert, light sensor, temperature sensor, intravaginal device, cattle

## Abstract

One critical point of dairy farm management is calving and neonatal first care. Timely calving assistance is associated with the reduction of calf mortality and postpartum uterine disease, and with improved fertility in dairy cattle. This study aimed to evaluate the performance and profitability of an intravaginal sensor for the prediction of stage II of labor in dairy farms, thus allowing proper calving assistance. Seventy-three late-gestating Italian Holstein cows were submitted to the insertion of an intravaginal device, equipped with light and temperature sensors, connected with a Central Unit for the commutation of a radio-signal into a cell phone alert. The remote calving alarm correctly identified the beginning of the expulsive phase of labor in 86.3% of the monitored cows. The mean interval from alarm to complete expulsion of the fetus was 71.56 ± 52.98 min, with a greater range in cows with dystocia (*p* = 0.012). The sensor worked correctly in both cold and warm weather conditions, and during day- or night-time. The intravaginal probe was well tolerated, as any cow showed lesions to the vaginal mucosa after calving. Using sex-sorted semen in heifers and beef bull semen in cows at their last lactation, the economic estimation performed through PrecisionTree™ software led to an income improvement of 119 € and 123 €/monitored delivery in primiparous and pluriparous cows, respectively. Remote calving alarm devices are key components of “precision farming” management and proven to improve animal welfare, to reduce calf losses and to increase farm incomes.

## 1. Introduction

Dairy farming globally is facing an increase in herd size, and the time farmers can spend observing animals is decreasing. One of the critical points of dairy farm management is represented by calving and neonatal first care. Calf death occurring during the first 48 h of life shows an incidence ranging from 2 to 20%, with 90% of calves being alive at the moment of parturition; this latter figure emphasizes that most of losses could be prevented [1]. Dystocia, defined as a difficult birth, is responsible for most calf losses due to the negative consequences that prolonged compression within the birth canal exerts on fetal homeostasis [2]. Calves born from dystocia may suffer from hypoxia, metabolic acidosis, or even from trauma and pain caused by forced extraction [1]. Dystocia is also related to an increased risk of trauma to the birth canal, postpartum uterine disease, reduced milk yield and prolonged calving to conception interval [3,4]. Dystocia accounts for 2–7% of all calving, with some variability related to farm, parity, breed, sire and calf sex [5]. Early recognition of the beginning of labor is important for prompt resolution of difficult births and ensures that the neonate calf is fed with an adequate quantity of good quality colostrum in the first hours of life [6]. Good colostrum management has been linked to optimal calf growth rate and even to heifers’ productive performance during their first and second lactation [7,8].

Timely calving assistance is directly associated with the reduction of calf mortality and postpartum uterine disease, and with improved fertility in dairy cattle [9,10]. However, continuous observation of late-gestation cows is time-consuming and the constant presence of an observer could also induce discomfort in periparturient animals leading to the release of catecholamines and interfering with the calving process [5]. Thus, identifying the beginning of parturition is challenging and most dystocia cases are faced in an emergency context, especially during night hours [10,11]. Furthermore, relatively low prevalence of dystocia, when compared with other postcalving diseases (i.e., mastitis), together with the low economic value of dairy male calves, lead to the farmers’ perception that difficult birth is a low-priority issue [1].

In the last decade, several sensors have been developed to improve herd management, as have methods to assess their performance [12,13]. Regarding the calving period, different strategies for imminent calving prediction have also been developed, such as automated remote sensors which can be fixed to the animal body to monitor the variation in behavior [14,15,16,17], rumination measures [18,19,20], body temperature [21,22] and tail movement [23,24] around calving. Information from these wearable sensors could be combined in machine-learning algorithms to refine their precision [25]. However, the prediction of parturition is still affected by an uncertainty interval of 6–12 h, and thus direct observation of ready-to-calve animals is still mandatory.

Automatic sensors for body temperature and motor activity generally show good sensitivity and specificity, but a certain number of false alarms are reported [24]. In fact, reliability of these systems could be affected by conditions which interfere with body temperature (fever, heat stress), the cow’s ability to move, feed and ruminate (lameness, uncomfortable flooring, systemic diseases), and tail movement (social interactions, regrouping, nuisance insects) [26]. A field trial concerning the performance of a tail movement sensor for calving prediction reported numerous events of detachment and severe skin lesions at the application site [23].

Promising results have been obtained by devices which could be placed within the vaginal canal, thus identifying the beginning of fetal expulsion (stage II of labor) with greater precision [10,21,27].

The objectives of this study were (i) to evaluate the sensitivity of an intravaginal sensor for predicting stage II of labor; (ii) to evaluate the time interval from the expulsion of the device followed by the generation of a calving alarm and the complete expulsion of the fetus; (iii) to evaluate the effect of a remote calving sensor on the profitability of rearing dairy and crossbred calves.

## 2. Materials and Methods

### 2.1. Animals and Husbandry

This study was conducted in a dairy farm located in Umbria Region, in Central Italy (42°95′ N, 12°39′ E). The herd’s mean composition was 160 Italian Holstein lactating cows, with an average milk production of 11,600 kg/cow/year. Milking cows were housed in adjacent freestall barns with cubicles, rubber mattresses and four milking robots (VMS, DeLaval S.p.A., San Donato Milanese, Italy). Animals had free access to feed bunk and water. A total mixed ration formulated for each productive group was distributed twice a day, between 04:00 and 05:00 and between 15:00 and 16:00. Heifers were submitted to the first artificial insemination around 15 months old and first calving was achieved at 24–25 months. Inseminations were performed on both natural or synchronized estrus, with heifers being inseminated with female sex-sorted semen. Pluriparous cows were inseminated with Holstein bull semen, while those at the end of their productive career were inseminated with beef bull semen. Pre-calving areas had multiple boxes with straw bedding. Pregnant animals were moved there 3 weeks before the expected calving.

### 2.2. Experimental Design

This study involved 73 Italian Holstein cows, and covered the period from February 2019 to January 2020. Every 15 days, heifers and cows in the precalving areas were randomly extracted through casual selection of their identification number. They were subsequently included in the experimental group and clinically evaluated twice a week for detecting premonitory signs of parturition, such as relaxation of pelvic ligaments, loosened cervical mucus plug, edema of the vulva and mammary gland. Once those signs were detected, the intravaginal device was applied. Briefly, scrub of the perineum and vulva were performed with diluted iodine solution (7.5% Povidone-Iodine solution, Betadin Meda Pharma S.p.A., Milano, Italy). Then, the device was immersed for 20 min in warm diluted iodine solution and inserted through a lubricated gloved hand into the vaginal cavity with the rounded extremity in contact with the external cervical os as we described in buffaloes [28].

Once the calving alert was received, the operators reached the calving area within 20 min, ensuring obstetrical evaluation to assess fetal presentation, position and posture, together with the degree of cervical dilatation. Calving assistance was carried out according to the recognized obstetrical procedures [29].

A degree of calving difficulty was assigned to each calving as follows:

0— Eutocia;
1— Prolonged expulsive phase with normal fetal presentation;
2— Slight dystocia with fetal abnormal presentation;
3— Moderate dystocia, foeto-maternal disproportion;
4— Severe dystocia such as uterine torsion and cervical stenosis.

Calves were submitted to Appearance, Pulse, Grimace, Activity and Respiration scoring (APGAR) as described by Vannucchi et al. [30]. Briefly, the following clinical parameters were considered and scored: mucous membranes color (0 = cyanotic; 1 = pale; 2 = normal), heart rate (0 = absent; 1 = bradycardia, <60 beats per minute or irregular; 2 = regular, >80 beats per minute), muscle tone (0 = flaccid; 1 = slight flexion; 2 = flexion), activity (0 = absent; 1 = some movement; 2 = active calf) and respiration (0 = absent; 1 = irregular < 24 respiration per minute; 2 = regular > 36 respiration per minute). Two liters of colostrum of good quality, that is with IgG content > 50 g/L and score > 22% on a Brix scale [8], was administered to calves within 2 h of birth.

### 2.3. Remote Calving Alarm Apparatus

The calving alarm system (OraNasco^®^, Kronotech s.r.l., Basaldella di Campoformido, Italy) consisted of a control unit and an intravaginal device, as we described previously [10].

**Figure 1:**
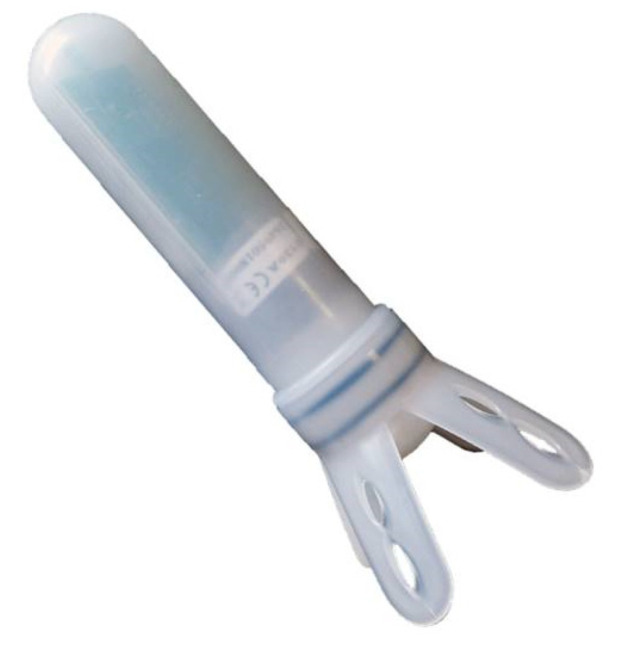
Representation of the intravaginal probe for the calving alarm system. The base works as an anchoring system while the cylindrical bin contains light and temperature sensors, together with a circuit board equipped with a transmitter. The probe is 174 mm in length, the cylindrical bin is 34 mm in diameter and the fin-shaped base is 78.5 mm in diameter. The rounded extremity contains a magnet for communication with the Central Unit.

The vaginal probe (60 g weight) is composed of an anchoring base and a cylindrical bin (Figure 1). The former is fin-shaped in order to secure the device to the vaginal wall, and the bin contains physical sensors such as light and temperature, together with a circuit-board and a transmitter. The case of the probe is made of low-density biocompatible polyethylene (LDPE, Riblene^®^, Versalis SpA S Donato Milanese Italy).

**Figure 2:**
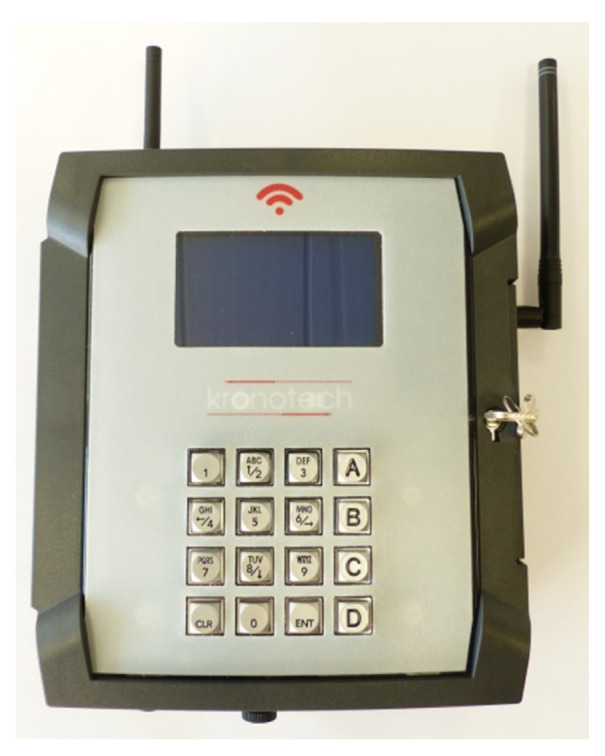
The Central Unit is able to manage up to six intravaginal devices. The Unit is composed of a screen, a keyboard and an internal housing which contains the circuit board, the slot for the cell phone Sim Card and a battery. To activate the probe, this is placed with the “magnet side” toward the red symbol 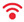.

**Figure 3:**
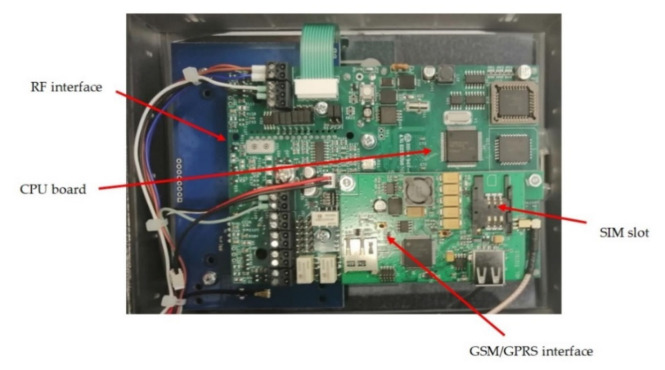
Circuit board housed within the Central Unit. RF interface is in charge of communication between the apparatus and the vaginal probes. The GSM/GPRS interface is in charge of communication between the Central Unit and mobile phones, by means of the mobile phone SIM card placed into the dedicated slot.

The Central Unit (Figure 2) can simultaneously manage up to six probes and should be placed within 30 m from gestating cows. Before insertion, the intravaginal device is activated through the Central Unit and a progressive number is attributed, so that when the alarm is received, the calving cow could be immediately recognized. The Central Unit is powered by +12 V through a transformer power supply but in case of energy network blackout the internal battery ensures a short interval of life (5 min on average), which is sufficient to send a warning through SMS. The apparatus (Figure 3) is equipped with a slot for the cell phone Sim card, thus it is able to communicate with user’s contacts by GSM/GPRS. The communication between the vaginal probes and the Central Unit is based on a 868 MHz radiofrequency; both of them can work within a temperature interval from −20 °C to +55 °C.

**Figure 4:**
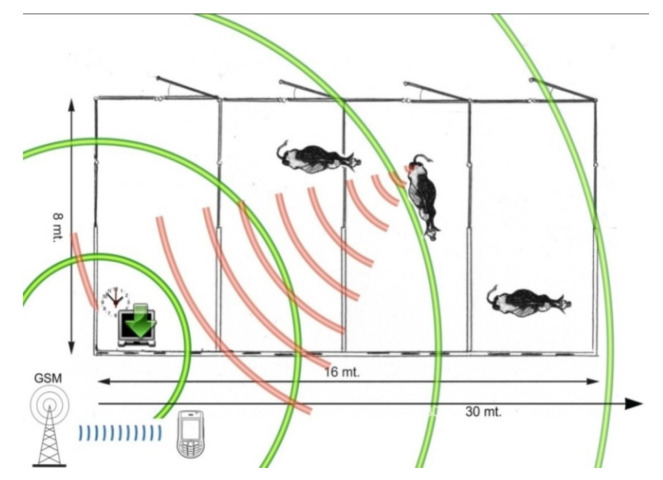
Schematic illustration of the remote calving alarm system. The Central Unit is able to simultaneously manage up to six intravaginal probes and should be placed within 30 m from gestating cows. At the beginning of stage II of labor the probe is expelled from the vaginal canal, leading to the activation of the radio signal, which is decoded by the Central Unit. The cell phone Sim Card within the Central Unit uses the GSM connection to send the alert to the user contacts recorded in the internal memory.

The temperature sensor of the intravaginal probe is set to recognize temperature gradients, while the light sensor can generate an output even in case of scarce brightness, such as at dusk or at night. When the device is activated, the internal board carries out a countdown for 60 min, which are enough for the insertion into the vaginal canal. During this period, the probe is in a suspended state and does not perform any control. After 60 min, the probe starts to check for light or a sudden change in temperature. If at least one of the two conditions is present, the probe switches to the ejected status and communicates the expulsion through a radio signal to the Central Unit, then it turns-off autonomously. The expulsion of the probe occurs when the fetal sacs or the fetus itself enter the birth canal, at the beginning of stage II of labor. When the Central Unit receives the expulsion signal from the intravaginal probe, this activates the GSM autodialer that sends an SMS and a phone call alert to user contacts (Figure 4).

### 2.4. Data Collection

For each cow enrolled, a database containing cow id, parity, estimated date of calving, date of alarm insertion, date and hour of calving alarm reception, and complete expulsion of the calf, together with calf sex and dystocia score was built in Excel™.

### 2.5. Statistical Analysis

Statistical analyses were carried out on statistical software XLSTAT statistical and data analysis solution rel.2021.2.2 (Addinsoft, New York, NY, USA). Student T-test for non-paired data was applied to compare means and χ^2^ test to compare frequencies; the α level was set at 0.05.

### 2.6. Decision-Tree Analysis

To assess the payoff of the probability of each possible calving outcome, based on the application or not of the sensor, a decision-tree analysis was performed by means of PrecisionTree™ software (ver. 8.1.0. 2020 Palisade Corp. Ithaca, NY, USA). The software determines the best decision to make at each decision node. Once the decision tree is complete, it creates a full decision analysis statistics report and its comparison with alternative decisions, as reported in studies in human and veterinary diagnostics [31,32]. The input values for this analysis are reported in Table 1.

In more detail, we assumed that the battery of the intravaginal probe could last for up to 30 deliveries. Thus expenses associated with the Sensor and Central Unit for each calving amounted to 10 €, which included costs calculated in our previous study together with a rounded flat rate represented by extra work, disposable gloves, scrub and other consumables [33]. Market values for dairy and crossbred calves were extrapolated from the Italian Institution for Agro–Food Market Services (ISMEA) [34]. The ratio for using female sex-sorted semen for artificial insemination in heifers or beef bull semen in adult cows was extrapolated by Bellingeri et al. [35]. The probability of having dystocia in cows carrying a purebred dairy or crossbred calf was reported by De Amicis et al. [36] and by Gafaar et al. [37], respectively. Mortality rates in monitored and unmonitored cows were those observed in our previous study [33].

## 3. Results

### 3.1. Data Description

**Figure 5:**
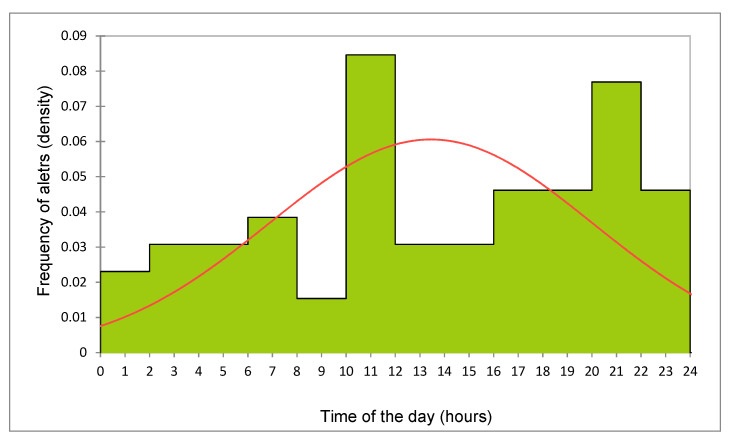
Distribution of phone alert reception for imminent calving, during the day, expressed as the proportion of calls within 24 h (density). The red line describes the fitted distribution of calls.

Table 2 describes the sensor response data, the mean interval from the application of the intravaginal device and calving, and from the phone alert and the complete expulsion of the fetus. In this study, the mean interval from the implantation of the alarm device to calving was 5.10 ± 3.58 days, except in one animal with prolonged pregnancy that calved a live calf 18 days after the insertion of the probe. Figure 5 shows the distribution of phone alerts received during the day. About one quarter of calving (24.6%) occurred between 00:00 and 06:00 and one-third (33.8%) between 22:00 and 06:00.

Data concerning the sensitivity of the remote calving alarm and the calving difficulty outcomes are summarized in Table 3. The sensors applied in 73 cows correctly reported an alarm before calving in 63 cases (Sensitivity: 86.30%). In 10 cows no alarm was received at the beginning of calving due to the failure of the GSM network in the area that occurred during the study period.

A dystocia was observed in 30.2% of the cases with positive alarm (19 cases), independently on the degree assigned. Prolonged duration of the expulsive phase (score = 1) was observed in 13.7% of monitored deliveries. Severe dystocia (score = 4) requiring a cesarean section for resolution did not occur during the study. All calves were born alive, and no calf death occurred within the first 48 h of life. The time interval between sensor application and calving was not statistically different for cows with dystocia (4.4 ± 2.6 d) compared with cows with eutocia (5.4 ± 3.9 d), even if numerically shorter (Table 2). Calving duration was statistically longer (*p* = 0.012) in cows with dystocia (80:16 ± 29:48 min) than in cows with eutocia (56:54 ± 33:39 min).

### 3.2. Decision-Tree Analysis

The decision tree analysis was carried out separately for primiparous and pluriparous cows. Indeed, when primiparous are considered, a larger number of female calves is expected, whereas pluriparous cows at the end of the production cycle are usually inseminated with beef semen. Moreover, the calf mortality rate is different in primiparous and pluriparous cows [1,36], and the cost analysis will thus be different.

**Figure 6:**
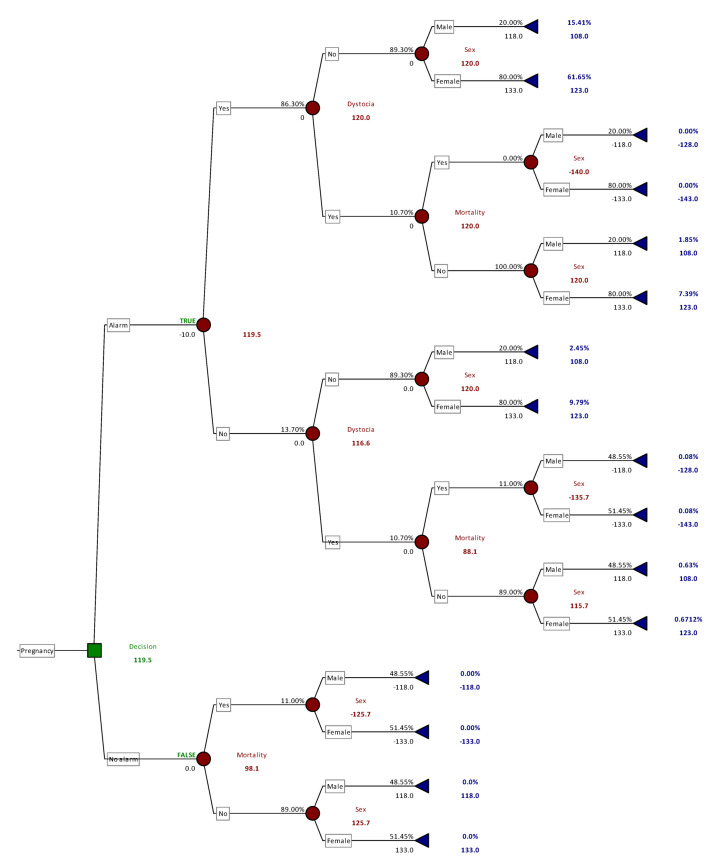
Decision-tree for the application of the calving sensor in primiparous cows. Green square identifies the decision to apply the sensor. Red dots represent the binary options, such as alarm sensitivity and dystocia outcome, based on data reported in Table 1. Black numbers on branches represent the probability of each chance (number over the branch), while the number below the branch represent the monetary value of the outcome (EUR). Red numbers within branches represent the monetary value of the combined outcome (EUR). Blue numbers at the end of the branches represent the probability and monetary value (EUR) of the whole branch.

**Figure 7:**
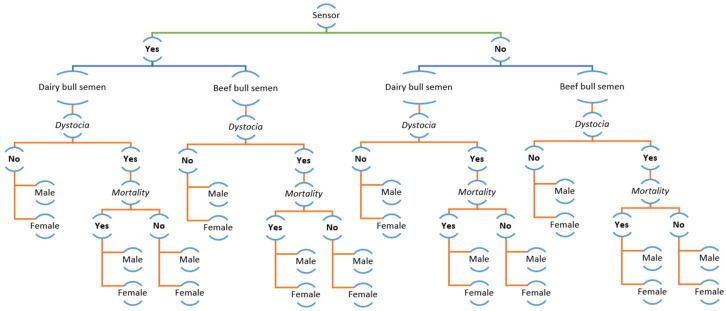
Summary of decision-tree for the application of calving sensor in pluriparous cows. Each point reported in the figure, such as dairy bull semen and dystocia, represents a binary option based on data reported in Table 1. Data cannot be represented due to the very large size of the figure.

Figure 6 describes in detail the outcome of the decision-tree analysis for primiparous cows. The input values and the outcomes are reported at each branch of the analysis. The detailed description of analysis for pluriparous cows, being more complex, was impossible to reduce to a readable figure. Therefore, Figure 7 reports all the potential branches and the outcome of the model (the detailed version is included in Supplementary Materials).

The results from the decision tree model showed that the application of the calving sensor in primiparous cows showed a positive outcome of EUR 119.5, when compared with the absence of the sensor. When pluriparous cows were considered, the estimated outcome gave a positive result of EUR 123.6.

A sensitivity analysis was also performed for both primiparous and pluriparous cows, varying the sensitivity of the sensor in a range ± 50%, as well as the frequency of dystocia in the range ± 25%, and the results showed that the changes in the outcome comprised small variations, being in the range of EUR 1–2.

## 4. Discussion

Calving assistance and the first neonatal care should be improved worldwide; however, predicting the beginning of parturition is still difficult. This study aimed to evaluate the performance of an intravaginal device for the identification of the expulsive phase of delivery, by analyzing the payback associated to improved calving assistance and first neonatal care in dairy farms.

The intravaginal device is made from a biocompatible material (Riblene^®^), also approved in EU for the manufacture of food containers; thus, no compounds were transferred to vaginal tissues. In the cow that calved 18 days following the insertion of the device, few sign of discomfort occurred, even if a slight catarrhal vaginal discharge during the last 3 days before calving was observed. We noticed a few heifers showing tail raising after insertion of the probe for up to 30 min, while no abnormal behavior was observed in the following days. The device was well tolerated, as any subject showed tearing, ischemic compression damage or hyperemia effects on the vaginal mucosa after calving.

A prolonged expulsive phase due to relative fetal oversize was included in the slight dystocia category. This was responsible for the apparent greater overall incidence of dystocia, but when accounting only for moderate and severe dystocia, a 13.33% prevalence was observed, in line with previous reports [5,38,39]. Choukeir et al. [40] used a similar intravaginal calving alarm device to identify the beginning of labor and found that delivery averaged 101.5 ± 92.5 min, which is greater than what was observed in this work. In their study, obstetrical assistance was provided only if no calving progression was assessed one hour after receiving the alarm, while in the present study, each cow was obstetrically evaluated within 20 min after receiving the alert. Thus, we immediately recognized and corrected fetal maldispositions, reducing the overall time for calving. Since the exact time of expulsion of the sensor was known from the time of the SMS and phone call reception, we could set 30 min intervals for evaluating calving progression. The identification of the beginning of calving leads to timely colostrum administration, thus ensuring calf growth stimulation, protection against neonatal diseases and improved calf welfare [7,39,40]. Average environmental temperatures in Umbria Region (Central Italy) range between −5 °C and +7 °C in winter, and between +24 °C and 34 °C in summer (https://tinyurl.com/z6sd52dc (accessed on 11 December 2021)). The device was used and worked correctly in both cold and warm weather conditions, and during day- or night-time. Some authors have used a similar intravaginal device [41] equipped with temperature sensors for detecting expulsion at the beginning of the expulsive phase in wild large ruminants. However, they reported that in a high temperature climate, the difference between internal and external temperature was sometimes not enough to generate the alert. Another intravaginal calving alarm device is the Medria Vel’Phone^®^ [21,24,40]. This system, which is equipped only with temperature sensors, can send two different alarms; the former is due to a physiological decrease in vaginal temperature around 48 h before calving, while the latter is due to the expulsion of the device at the beginning of labor. Authors of these studies reported no lacking alarms; however, Choukeir et al. [40] observed false positive 48 h alerts during summer, probably due to heat stress. The sensor used here could monitor both temperature and light, however, thus ensuring that at least one physical indicator would be effective.

The economic evaluation presented here defined a model as close as possible to the usual management practices in modern dairy herds. Indeed, it considered the use of sex-sorted semen and beef bull semen for inseminations in heifers and last-lactation cows, respectively. Our estimation of the economic benefit of applying a remote calving alarm system leads to an income of EUR 119 and EUR 123/monitored delivery in primiparous and pluriparous cows, respectively. This amount is similar to what reported by McGuirk et al. [42] for slight difficult in calving followed by calf loss. In cases of severe dystocia, they reported greater losses up to GDP 400; however, they also considered veterinary intervention costs and losses due to reduced fertility and culling in cows affected by dystocia. Similarly, Mahnani et al. [43] estimated that calf death in dairy farms accounted for a total loss ranging from USD 700 to USD 1100, but this estimation also considered long-term consequences of stillbirth such as replacement heifer costs. The estimation provided in our study examined only the probability of dystocia and calf loss together with the value of the live calf as an income and was not dependent on long-term consequences such as milk production and fertility, as provided for in our previous study [33]. Thus, the evaluation of the return of investment could rely on short-term estimation. Moreover, in the previous report the direct effect of dystocia could not be evaluated, while in the present study dystocia and its effect on calf viability and farm economy were accounted for, with greater external validity. The economic effort required to purchase these devices is usually limited and can be quickly repaid by the gain of live calves as shown in this study, supporting previous evaluations [40].

Machine-learning algorithms, which use behavioral data from activity sensors to predict calving [25] still have limited precision and need the combination of various sensors, which is not always economically sustainable. The system described above specifically identifies the beginning of fetal expulsion, thus optimizing personnel presence in the calving area at the right time. This led to prompt calving and neonatal assistance and colostrum feeding within the first hours of life.

## 5. Conclusions

The remote calving alarm used in this study correctly identified the beginning of the expulsive phase of labor in 63 out of 73 of the monitored cows, for an overall sensitivity of 86.3%. The mean interval from alarm reception to the complete expulsion of the fetus was 71.56 ± 52.98 min, with greater range in cows with dystocia. Considering the use of female sex-sorted semen in heifers and a certain amount of beef bull semen in adult cows, our estimation of the economic benefit of remote calving monitoring leads to an income gain of EUR 119 and EUR 123/monitored delivery in primiparous and pluriparous cows, respectively. The sensor used here could monitor both temperature and light, thus ensuring that at least one physical indicator would be effective even in the case of heat stress, and worked correctly in both cold and warm weather conditions, as during day- or night-time. The device was well tolerated, as any cow showed tearing, ischemic compression damage nor hyperemia to the vaginal mucosa after calving.

Remote calving alarm devices are fundamental to optimize farmers’ workload and to improve their quality of life, by warning of an ongoing delivery and avoiding the need for continuous monitoring of pregnant animals. These sensors are key components of “precision farming” management and proven to improve animal welfare, to reduce calf losses and to increase farm incomes.

## 6. Patents

OraNasco^®^, patent number: 0001405187–12/20/2013–WIPO: 10UD2011A000062, “Sistema di rilevamento e di allerta del parto incipiente negli animali domestici e di allevamento”.

## Figures and Tables

**Table 1:** Market prices and epidemiology parameters applied in the decision-tree analysis.

Variable	Value	Source
Sensor and Central Unit cost (per calving)	10 €	[33]
Value of a male dairy calf	50 €	[34]
Value of a female dairy calf	100 €	
Value of a male crossbred beef calf	142 €	
Value of a female crossbred beef calf	219 €	
Probability of having a male calf/no sex-sorted semen	0.485	This paper
Probability of having a female calf/no sex-sorted semen	0.516	
Probability for using sex-sorted semen in primiparous cows	0.85	[35]
Probability for using beef semen in pluriparous cows	0.15	
Sensitivity of the remote calving sensor	0.863	This paper
Probability of dystocia with a dairy calf	0.0375	[36]
Probability of dystocia with a crossbred calf	0.085	[37]
Mortality in primiparous cows with sensor	0	[33]
Mortality in pluriparous cows with sensor	0.017	
Mortality in primiparous cows without sensor	0.11	
Mortality in pluriparous cows without sensor	0.127	

**Table 2:** Sensor response data. The overall interval from sensor application and calving, for eutocia or dystocia. The overall interval from calving alarm to the complete expulsion of the fetus, and in case of eutocia or dystocia.

Parameter	Animals (*n*)	Mean ± Std. Dev.	1st Quart.	Median	3rd Quart.	Min	Max
Time from sensor application and calving (d)	63	5.10 ± 3.58	3.0	5.0	6.0	0	18.0
Eutocia	44	5.40 ± 3.90	3.0	5.0	7.0	0	18.0
Dystocia	19	4.40 ± 2.60	2.5	4.0	6.0	0	10.0
Interval between alarm and calving (min)	63	72 ± 53	34	62	93	15	146
Eutocia	44	57 ± 33 ^a^	33	60	73	15	146
Dystocia	19	80 ± 29 ^b^	64	88	103	24	126

^a,b^ Significant difference was observed between eutocia and dystocia (*p* = 0.012).

**Table 3:** Sensitivity of sensor and frequency of calving difficulty. 0—eutocia; 1—prolonged expulsive phase with normal fetal presentation; 2—slight dystocia with fetal abnormal presentation, resolution by manual correction; 3—moderate dystocia, foeto-maternal disproportion; 4—severe dystocia such as uterine torsion and cervical stenosis.

Categories	Number of Cattle per Category (*n*)	Relative Rate per Category (%)	Lower Bound of Frequencies (95%)	Upper Bound of Frequencies (95%)
Alarm	63	86.30	78.41	94.20
No alarm	10	13.70	5.81	21.59
Calving difficulty:				
Score 0	44	60.27	49.05	71.50
Score 1	10	13.70	5.81	21.57
Score 2	5	6.85	1.06	12.64
Score 3	4	5.48	0.26	10.70
Score 4	0	0	0	0

## Data Availability

The data presented in this study are available on request from the corresponding author.

## Supplementary Materials

The following are available online at https://www.mdpi.com/article/10.3390/s21248348/s1, Figure S1: Decision-tree for the application of calving sensor in pluriparous cows. Green square identifies the decision to apply the sensor. Red dots represent the binary chances, such as alarm sensitivity and dystocia outcome, based on data reported in Table 1). Black numbers on branches represent the probability of each chance (number over the branch), while number below branch represent the monetary value of the outcome (EUR). Red numbers within branches represent the monetary value of the combined outcome (EUR). Blue numbers at the end of the branches represent the probability and monetary value (EUR) of whole branch.
