# Supplementary material for: Assessment of Sensitivity and Profitability of an Intravaginal Sensor for Remote Calving Prediction in Dairy Cattle
Source: Sensors (Basel). 2021 Dec 14;21(24):8348. doi: 10.3390/s21248348 (PMC8706507; doi:10.3390/s21248348)
Supplement: Supplementary file 1 [file sensors-21-08348-s001.zip › sensors-1459681-supplementary.pdf]

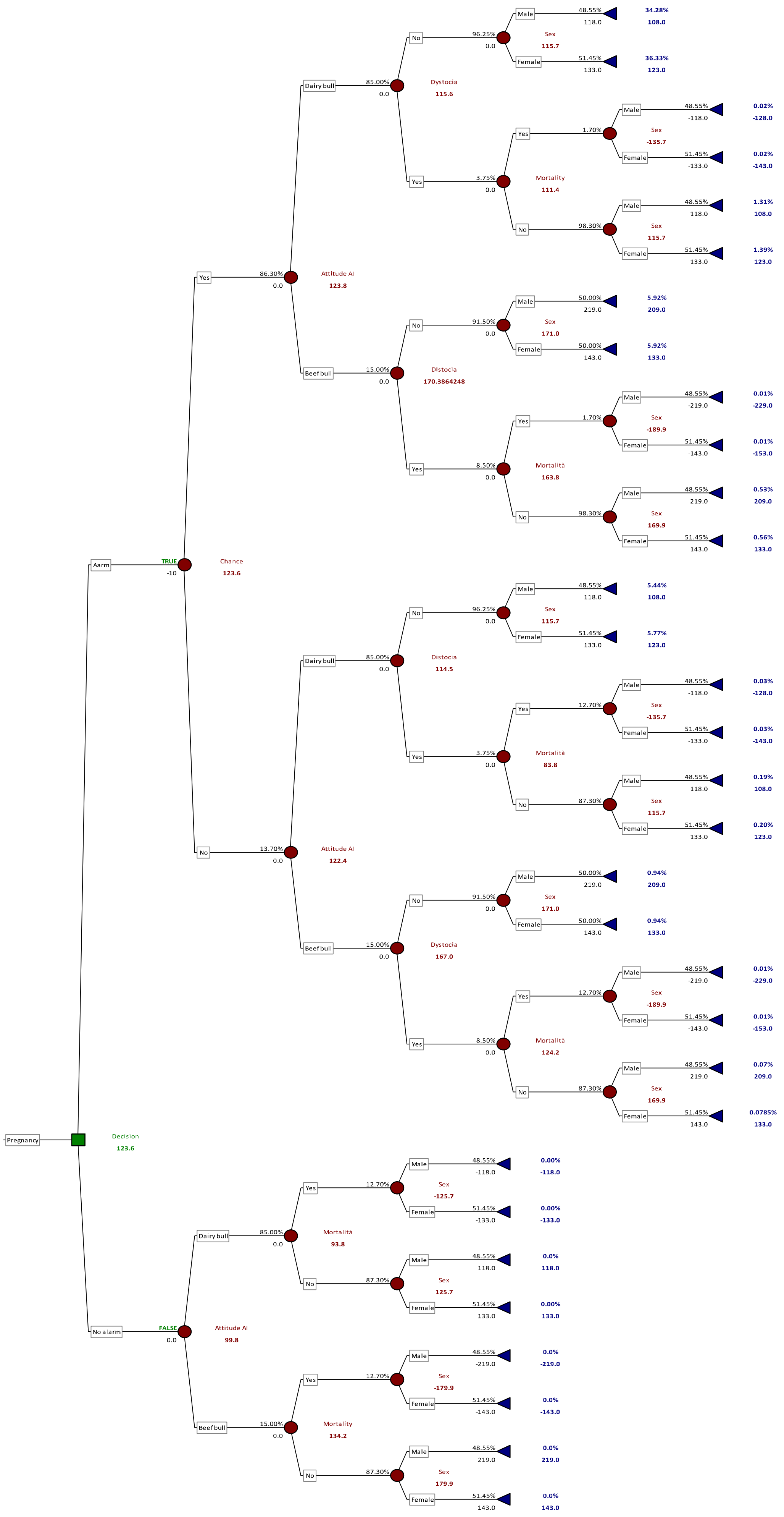

Figure S1. Decision-tree for the application of calving sensor in pluriparous cows. Green square identifies the decision to apply the sensor. Red dots represent the binary chances, such as alarm sensitivity and dystocia outcome, based on data reported in Table 1). Black numbers on branches represent the probability of each chance (number over the branch), while number below branch represent the monetary value of the outcome (EUR). Red numbers within branches represent the monetary value of the combined outcome (EUR). Blue numbers at the end of the branches represent the probability and monetary value (EUR) of whole branch.
